# Prevalence and correlates of intimate partner violence against women in conflict affected northern Uganda: a cross-sectional study

**DOI:** 10.1186/s13031-019-0219-8

**Published:** 2019-07-30

**Authors:** Eleanor Black, Heather Worth, Susan Clarke, James Henry Obol, Peter Akera, Agnes Awor, Mike Sevenska Shabiti, Helen Fry, Robyn Richmond

**Affiliations:** 1 University of New South Wales – Faculty of Medicine, School of Public Health and Community Medicine, Kensington, Australia; 2 Gulu University – Faculty of Medicine, School of Public Health, Gulu, Uganda

**Keywords:** Intimate partner violence, Conflict affected, Northern Uganda, Alcohol

## Abstract

**Background:**

Intimate partner violence (IPV) is an important public health issue as it impacts negatively on health, economic and development outcomes. In conflict affected northern Uganda, IPV prevalence is high and additional context-specific risk factors exist. People residing in this region have been displaced, exposed to war and violence, and had livelihoods destroyed. There are few studies examining IPV in this setting. In this study we aim to further understand the prevalence of IPV towards women and its associations in conflict affected northern Uganda.

**Methods:**

This was a cross-sectional, behavioural survey designed to capture quantitative information related to experiences of IPV among women living near two health clinics in rural northern Uganda. There were 409 women who participated in the survey. Data were analysed using logistic regression.

**Results:**

High rates of emotional, physical and sexual IPV were found; 78.5% of women had experienced at least one type of IPV, and slightly more than half of the participants had experienced IPV in the 12 months prior to the survey. Many women felt that IPV was justified in certain situations. Significant determinants of IPV included alcohol abuse by the male partner (OR 2.22, 95% CI 1.34–3.73); partner having been in a physical fight with another man (OR 1.90, 95% CI 1.12–3.23); controlling behaviours by the male partner (OR 1.21, CI 1.08–1.36). and younger age of the woman (OR 0.95, 95% CI 0.92–0.98). Educational level was not independently associated with IPV.

**Conclusions:**

Our findings show that IPV is a significant issue in conflict affected northern Uganda, and attitudes that normalise and justify IPV are common. Alcohol abuse among young men in northern Uganda is highly prevalent and strongly associated with IPV towards women, as are controlling behaviours exhibited by the male partner. Interventions to reduce alcohol consumption among men in this region are likely to have important benefits in reducing the prevalence of IPV, and attitudes and behaviours that support IPV need to be further understood and addressed. Many women in conflict affected northern Uganda likely have additional risk factors for IPV related to previous exposure to war violence, however this was not directly measured in the present study. Further research into IPV in northern Uganda, and its relationship to exposure to conflict, is greatly needed.

## Background

The term ‘intimate partner violence’ (IPV) describes abusive behaviours between partners in an intimate relationship and can broadly be classified as abuse of a physical, emotional or sexual nature [1, 2]. Women are much more likely to be victims of IPV than men, globally as well as in sub-Saharan Africa (SSA) [3]. IPV violates human rights and adversely affects economic and social development [4, 5]. It impacts negatively on physical, mental, sexual and reproductive health, and is associated with higher rates of low birth weight babies, unsupervised abortions, depression, alcohol abuse, and HIV infection [6–8]. The body of research on IPV prevalence and its health effects is limited but has increased substantially over the past decade through household and national health surveys, such as the Demographic and Health Surveys (DHS) [9–11] and population-based cross-sectional data [6].

The World Health Organization’s (WHO) systematic review on IPV, using aggregated global and regional prevalence estimates, reported a worldwide IPV prevalence of 30%, which was higher (37%) in WHO African regions [6]. Within SSA, lifetime prevalence rates for IPV vary but are consistently high; the WHO 2006 multi-country study on violence against women, using data from 10 countries and 15 study sites, reported a lifetime prevalence of physical and/or sexual IPV of 71% in Ethiopia, 56% in Tanzania and 36% in Namibia [12]. The Kenya 2014 DHS found that 45% of women have experienced physical violence since age 15 years [11]. The Uganda 2016 DHS found that 58.4% of married women reported ever having experienced emotional, physical or sexual violence from a spouse, and 39.6% had experienced it within the past year [9].

We carried out our study among women living near two health clinics in northern Uganda. IPV in northern Uganda needs to be understood in the context of a long history of social, economic and political upheaval and exclusion from the centre of the country, which intensified during 1986–2006 when the local population endured bouts of severe violence from both the Lord’s Resistance Army (LRA) and from government forces [13] engaged in the civil war that engulfed the region. The war caused major economic disruption and over 500,000 Acholi people were displaced and suffered the loss of means of production, household assets and other investments [14]. They endured profound emotional and psychological stress. Most of the children did not attend school. The majority of northern Ugandans had inadequate access to basic services including healthcare and the prevalence rates of sexually transmitted infections and HIV had increased when compared with the rest of Uganda [14]. The two health clinics where we carried out our research, Awach and Lalogi, are situated in the middle of the most severely conflict affected districts.

Violent conflict adds to an array of traditional determinants of poverty, such as education, livelihoods and health, and, in so doing, limits future progress in human development. The effects can persist for a long time after the conflict has ended [15]. Life in the conflict affected setting of northern Uganda is characterised by factors that create instability, including ongoing land disputes, displacement, an erosion of traditional Acholi culture and values, and post-traumatic psychological effects [19]. Exposure to war-related events and experiencing violence in adulthood are recognised risk factors for IPV [4, 16], and studies have demonstrated that rates of IPV are high in conflict affected regions [12, 16, 17]. Many of the women who lived through the conflict in northern Uganda did not have the benefit of recognised protective factors for IPV such as formal education and economic empowerment [7, 21]. Other recognised risk factors for IPV observed globally include younger age of the woman, cohabitation, having outside sexual partners, alcohol abuse by either partner, controlling behaviours exhibited by the male partner, and attitudes that justify IPV [3, 4, 7, 8, 16]. Many of these risk factors, in particular alcohol abuse among men, and justification of IPV, are highly prevalent in northern Uganda [4, 8, 9].

In the Acholi region of Uganda, where Awach and Lalogi are situated, the lifetime prevalence for IPV was 59.9% according to the 2016 Uganda DHS [9]. However, research into factors that contribute to IPV in the conflict affected northern Uganda setting is limited. Existing research has mainly focused on physical and sexual IPV, and there is a paucity of research on emotional IPV in this setting. Most studies on IPV in northern Uganda were conducted during or in the immediate aftermath of the conflict [20, 22, 23], and found high rates of IPV among women living in internally displaced persons camps (IDPs) in northern Uganda [17, 20, 23]. Fewer studies have examined more recent patterns of IPV among Acholi women in northern Uganda, at a time when the majority of internally displaced people have left the camps and re-settled. The aim of our research is to examine the prevalence of IPV against women in conflict affected northern Uganda, and to evaluate factors among women and men that increase the likelihood of IPV. In our study we demonstrate that IPV is extremely prevalent among women of all ages in northern Uganda, more than a decade after the 2006 ceasefire. Understanding the factors that have allowed IPV to continue unabated in the conflict affected setting of northern Uganda is critical to addressing this issue.

## Methods

### Study design and aims

The study was a cross-sectional behavioural survey designed to capture quantitative information from women related to their experiences of IPV. The study took place at two health clinics in northern Uganda, in Gulu and Omoro districts, where the local population had been severely affected by the conflict.

### Study questionnaire

The study questionnaire was developed using a validated questionnaire (the Woman’s Questionnaire) from the Uganda 2011 DHS [10]. We adapted this questionnaire to capture information relevant to IPV and women’s wellbeing. The questionnaire we used included all components of the domestic violence module as well as questions on the background characteristics of respondents and their partners (educational attainment, literacy, employment status, marital status); fertility levels and family planning (number of children born and currently living, access to and use of contraception); sexually-transmitted diseases (STDs) and HIV-related knowledge, attitudes and behaviours; and indicators of women’s empowerment (attitudes towards IPV). The survey included questions relating to attitudes and behaviours around IPV. Women were asked about whether their partner exhibited certain controlling behaviours (maximum number of behaviours seven), whether they felt that men had a ‘good reason’ to hit his wife in particular situations (maximum number six), whether they felt that there were ‘good reasons’ for refusing sex with their partner (maximum number four), and whether they agreed with six different statements relating to intimate aspects of relationships (maximum number six). The mean number of instances in which women agreed with these statements/scenarios was analysed. The domestic violence module of the Uganda DHS is based on a modified version of the Conflict Tactics Scale, which asks about a range of specific acts of physical, emotional and sexual violence. A key advantage of this approach is that it is less likely to be affected by different understandings of what constitutes ‘violence’ and gives the respondent multiple opportunities to disclose any experience of violence [10].

The questionnaire was structured for the tablet format of delivery. All women aged 16 and over were eligible to participate. Participants had the option of doing the survey alone (one of the advantages of the tablets is that they can afford privacy) or the survey being interviewer-administered. Due to very low levels of literacy all surveys were interviewer-administered.

### Sample size estimation and sampling methods

Data were collected over a period of two weeks in November 2017 by the University of New South Wales (UNSW) and Gulu University staff and students, reaching the desired sample size in that period. We calculated our sample size based on a key indicator of women’s health: the proportion of rural women who had used family planning in the past year. This key indicator has sufficient statistical power to detect differences in other key indicators by factors such as marital status. A prevalence estimate of 67% was obtained from the 2011 Uganda DHS [10] and a web-based calculator of sample size estimate was used. We estimated a sample size of 350 women aged 16 years and over attending either Awach Health Centre Level IV in Gulu district or Lalogi Health Centre Level IV in Omoro district. Consecutive sampling was used for all eligible women attending the health clinics.

### Data management and analysis

The instrument was administered through Windows- based tablets. QDS (Questionnaire Development System) software by NOVA Research Company was used for programming and hosting the questionnaire on each Tablet, as well as for transforming the data into SPSS. Data were administered by UNSW, updated daily from the field in a safe format and stored securely on a server at UNSW. Hard copy versions of the questionnaire were taken into the field as a backup.

We used SPSS 25 to perform the analysis. First we described the baseline characteristics of the total sample and exposure to IPV. Then we used *t-*test or cross-tabs to compare those participants exposed to IPV and those not exposed to IPV. We performed a multivariable logistic regression using the dependent outcome variable of participant’s experience of IPV in the previous 12 months and seven independent variables comprising: participant reporting seeing her partner drunk on most days, participant having knowledge that her partner had been involved in a physical fight with another man, participant’s age, partner’s age, partner refusing to use or stopping her using contraception, partner’s controlling behaviours, participant’s education and partner’s concurrent relationships with other women. Some of these independent variables were chosen based on t-tests and chi-square tests showing they were reported significantly more frequently among women with IPV exposure compared to women without exposure (Table 1), and additional variables were chosen because they were frequently reported as risk factors for IPV by other studies. These variables were measured using validated questions from the Uganda DHS described earlier.

### Ethical considerations

To check that protocols relating to sampling, data collection, and ethical procedures were being adhered to, the students were trained to follow protocols. Ethical approval was granted by the University of New South Wales Human Research Ethics Committee (HREC HC17795) and the Gulu University Research Ethics Committee (GU REC). Each participant provided written informed consent before participating in the study. The surveys were conducted privately with each participant and the information collected can only be accessed by principal investigators. Women were informed about women’s health services available to them at the local clinic and advised to speak with local health workers if any issues arose for them after completing the survey.

## Results

The study team interviewed 409 women who had a mean age of 33 years (Table 1). There were 80% of women who had attended school, and 26% reported that they were able to read. The majority (77.5%) of women were currently living with a male partner, most commonly (78.5%) dwelling with his relatives. HIV prevalence was high at 13%, and more than 70% of women had been tested for HIV in the previous 12 months. Partners had a mean age of 38.3 years and most (71%) were subsistence farmers. Demographic characteristics were significantly different between those women who had and had not experienced IPV in the previous 12 months. Women who had experienced IPV in the last 12 months were younger compared with women who had not experienced IPV in the last 12 months (mean age 31.4 vs. 35.0, *p* = 0.01) and were significantly more likely to have obtained some primary education (71.9% vs. 58%, *p* = 0.001). Their partners were younger (mean age 37.1 vs. 40.2, *p* = 0.028) and more likely to drink daily (40.8% vs. 24.3%, *p* = 0.041).

**Table 1:** Demographic characteristics of women who have and have not experienced IPV in last 12 months

	Total participant women	Experience of IPV in last 12 months	No experience of IPV in last 12 months	P*
Number of women	409	228 (55.7)	181 (44.3)	
Age of women (mean, SD)	32.98 (11.5)	31.35 (9.79)	35.03 (12.12)	0.01
Number of children per woman (mean, SD)				
Living	4.29 (2.36)	4.29 (2.39)	4.30 (2.34)	0.983
Dead	0.54 (1.16)	0.49 (0.94)	0.60 (1.39)	0.319
Literacy (number, %)				
Able to read	107 (26.2)	60 (26.3)	47 (26)	0.936
Able to write	123 (30.1)	68 (29.8)	55 (30.4)	0.902
Highest education level (number, %)				
None	79 (19.3)	31 (13.6)	48 (26.5)	0.005
Some primary	269 (65.8)	164 (71.9)	105 (58)	0.001
Completed primary	48 (11.7)	28 (12.3)	20 (11.0)	0.038
Completed secondary or further	12 (2.9)	3 (1.3)	8 (4.4)	0.447
Ever used contraception (number, %)	270 (66)	161 (70.6)	109 (60.2)	0.052
Women aged 16 to 49 (number, %)	375 (91.69)	220 (58.7)	155 (41.3)	0.0005
Currently using contraception (number, %)	135 (36)	82 (37.3)	53 (34.2)	0.585
HIV test in last 12 months (number, %)	291 (71.1)	169 (74.1)	122 (67.4)	0.234
HIV positive (number, %)	53 (13)	33 (14.8)	20 (11.4)	0.409
Marital status (number, %)				
Currently married	191 (46.7)	109 (47.8)	82 (45.3)	0.0005
Living with a man, not married	126 (30.8)	84 (36.8)	42 (23.3)	0.003
Regular sexual partner, living apart	22 (5.4)	13 (5.7)	9 (5.0)	0.755
Not currently involved in sexual relationship	69 (16.9)	22 (9.6)	47 (26)	0.0005
Age of partner (mean, SD)	38.27 (12.35)	37.06 (11.43)	40.15 (13.49)	0.028
Dwelling with partner’s relatives in current or most recent relationship (number, %)	321 (78.5)	188 (82.5)	133 (73.5)	0.175
Partner’s occupation (number, %)				
Professional	28 (6.8)	17 (7.5)	11 (6.1)	0.096
Semi-skilled	37 (9.0)	16 (7.0)	21 (11.6)	0.165
Military/police	18 (4.4)	7 (3.1)	11 (6.1)	0.152
Other	8 (2.0)	4 (1.8)	4 (2.2)	0.589
Unskilled/manual	292 (71.4)	181 (79.4)	111 (61.3)	0.895
Partner’s alcohol intake (number, %)				
Daily	137 (33.5)	93 (40.8)	44 (24.3)	0.041
Once or twice per week	56 (13.7)	29 (12.7)	27 (14.9)	0.334
One to three times per month	15 (3.7)	7 (3.1)	8 (4.4)	0.378
Less than once per month	9 (2.2)	6 (2.6)	3 (1.7)	0.581
Never	173 (42.3)	90 (39.5)	83 (45.9)	0.043

*T-test for quantitative and χ^2^ for categorical data, testing difference between women who had and had not experienced IPV in previous 12 months

The participant women had high levels of lifetime exposure to IPV (78.5%) and more than half of them had been exposed to IPV in the previous 12 months (55.7%) (Table 2). There were 114 (27.9%) women who reported that a partner had hit them with his fist or with something else that hurt them, with around half having experienced this in the last 12 months (*N* = 60, 14.7%) (Table 2).

**Table 2:** Prevalence of intimate partner violence (emotional, physical and sexual) experienced by women in the past year

IPV type	Last 12 months *N* (%)
Emotional	
Insulted me and made me feel bad about myself	134 (32.8)
Belittled or humiliated me in front of other people	62 (15.2)
Did things to scare or intimidate me on purpose	109 (26.7)
Threatened to hurt me or someone I care about	74 (18.1)
Physical	
Slapped me or threw something at me that could hurt me	104 (25.4)
Pushed me or shoved me or pulled my hair	65 (15.9)
Hit me with his fist or with something else that could hurt me	60 (14.7)
Kicked me, dragged me or beat me up	69 (16.9)
Choked or burnt me on purpose	33 (8.1)
Threatened to use or actually used a gun, knife or other weapon	29 (7.1)
Sexual	
Physically forced me to have sexual intercourse when I did not want to	58 (14.2)
I had sexual intercourse when I did not want to because I was afraid of what he might do	89 (21.8)
Forced me to do something sexual that I found humiliating	30 (7.2)

Physical and emotional abuse had similar frequencies for lifetime experience (almost two-thirds), and for the last 12 months (more than one third), while sexual abuse was less frequent with almost half of the women experiencing sexual abuse from a partner in their lifetime (Table 3). Almost 30% of women reported that a partner had physically forced them to have sexual intercourse when they did not want to (*N* = 118, 28.9%), while 58 (14.2%) told us that they had been physically forced by their partner to have intercourse in the last 12 months.

**Table 3:** Women’s experiences of intimate partner violence in their lifetime and in the last 12 months

Intimate partner violence	Lifetime experience N (%)	Last 12 months *N* (%)
Physical abuse	250 (61.1)	143 (35)
Emotional abuse	265 (64.8)	175 (42.8)
Sexual abuse	192 (46.9)	115 (28.1)
Any intimate partner violence	321 (78.5)	228 (55.7)

Attitudes and behaviours around intimate partner violence are shown in (Table 4). The mean number of positive responses for ‘good reasons for a man to hit his wife’ was 2.84 (SD 1.5) per woman. This result included 155 (37.9%) women agreeing that in their opinion a man had a good reason to hit his wife if she did not complete her household work to his satisfaction. The mean number of controlling behaviours reported by women was 3.44 (SD 2.1) per woman. There were 170 (41.6%) of women who agreed that their partner tried to keep them from seeing their friends and 161 (39.4%) who agreed that their partner ignored them and treated them indifferently. Women’s attitudes to intimate relationships were explored, which revealed that 80% of women agreed with the statements that ‘a good wife obeys her husband even if she disagrees’ (*N* = 328, 80.2%) and that ‘it is important for a man to show his wife who is the boss’ (*N* = 333, 81.4%).

**Table 4:** Attitudes and behaviours around intimate partner violence

	Woman’s opinion number of ‘good reasons’ for a man to hit his wife	Woman’s report of number of controlling behaviours by partner	Woman’s opinion of number of good reasons to refuse sex with her partner	Woman’s attitudes to aspects of intimate relationships
Mean	2.84	3.44	2.52	3.71
Std. Deviation	1.55	2.06	1.18	1.27
Minimum	0	0	0	0
Maximum	6	7	4	6

Woman’s agreement with ‘husband has a good reason to hit his wife’, max 6 (she does not complete her household work to his satisfaction, she disobeys him, she refuses to have sex with him, she asks him whether he has other girlfriends, he suspects that she is unfaithful, she is unfaithful

Number of controlling behaviours by partner, max 7 (tries to keep woman from seeing friends, tries to restrict contact with birth family, insists on knowing whereabouts, ignores her/treats indifferently, gets angry if she speaks with another man, is often suspicious of unfaithfulness, expects to be asked permission to seek healthcare)

Number of ‘good reasons’ given by woman to refuse sex with partner, max 4 (if she does not want he, if he is drunk, if she is sick, if he mistreats her)

Woman’s attitude to aspects of intimate relationships, max 6 (a good wife always obeys her husband even if she disagrees, family problems should only be discussed with people in the family, it is important for a man to show his wife who is the boss, a woman should be able to choose her own friends even if her husband disapproves, it’s a wife’s obligation to have sex even if she doesn’t feel like it, if a man mistreats his wife, others outside of the family should intervene

Logistic regression in SPSS excludes cases with missing values on any of the independent variables leaving 386 women available for analysis. There were 226 women who had experienced IPV in the past 12 months and 160 who had not experienced IPV in the last 12 months. Our logistic regression model was statistically significant, χ^2^ (7, *N* = 386) = 57.63, *p* < 0.001, showing that the model was able to distinguish between participants who had and had not experienced IPV in the previous 12 months. The model was able to explain between 14.6% (Cox and Snell R square) and 19.7% (Nagelkerke R squared) of the variance in IPV status, and correctly classified 68.7% of participants. Table 5 shows results from bivariable and multivariable logistic regression analyses. Five of the independent variables (partner being drunk daily, participant having knowledge that her partner had been involved in a physical fight with another man, partner’s controlling behaviours, participant’s age and participant’s parity) made a statistically significant contribution to the risk of experiencing IPV in the last 12 months. The strongest predictor was seeing partner drunk on most days in the last 12 months, with an adjusted OR (aOR) of 2.22 (95% CI 1.33–3.73), indicating that a woman whose partner had been drunk on most days had almost twice the odds of having experienced IPV in the last 12 months compared to a woman whose partner had not been drunk on most days. Other significant predictors included the partner being involved in a physical fight with another man (aOR 1.90, 95% CI 1.12–3.23) and partner’s controlling behaviours (aOR 1.21, 95% CI 1.08–1.36). The aOR for woman’s age was 0.95 (95% CI 0.92–0.98), indicating that for every additional year of age the odds of experiencing IPV in the previous 12 months decreased by 0.95, when other factors in the model were controlled for. Although statistically significant on both bivariable and multivariable analysis, the OR for woman’s age was close to 1 in both analyses, indicating very little effect. For every additional child born to a participant, the odds of experiencing IPV in the last 12 months increased by 1.13 (95% CI 1.01–1.28). Partner’s sexual relationships with other women while also having a sexual relationship with the respondent was significantly associated with IPV on bivariable analysis (unadjusted OR 1.59, 95% CI 1.07–2.35) but this association did not remain when other variables in the model were controlled for. Completion of primary level education did not independently contribute to the model.

**Table 5:** Unadjusted and adjusted odds ratios with 95% confidence interval (CI) for factors associated with experience of intimate partner violence in previous 12 months

	Unadjusted		Adjusted*		Wald’s test
	OR	95% CI	OR	95% CI	Chi-sq *p*-values
Partner drunk most days in last 12 months	2.45	1.61–3.73	2.22	1.33–3.73	0.002
Partner has been in a physical fight with another man	2.67	1.68–4.24	1.90	1.12–3.23	0.017
Controlling behaviours by partner	1.32	1.19–1.47	1.21	1.08–1.36	0.001
Partner sexual relationship with other women concurrently	1.59	1.07–2.35	1.29	0.81–2.06	0.279
Woman’s parity	1.15	1.14–1.35	1.13	1.01–1.28	0.041
Woman’s age	0.97	0.96–0.99	0.95	0.92–0.98	< 0.001
Woman completed primary school	2.30	1.39–3.80	0.97	0.48–1.95	0.932
Constant			1.55		0.368

*Adjusted for all variables in table

## Discussion

The prevalence of IPV was extremely high in our study population; close to 80% of women had experienced at least one type of IPV in their lifetime, and 55% of women had experienced IPV within the past 12 months. Another survey of women in conflict affected northern Uganda also found high IPV prevalence rates of up to 80% [4], while comparably lower rates of 15 to 30% have been reported in relatively peaceful Ugandan regions [16, 18]. The relationship between prior exposure to violence, displacement, and high rates of IPV against women has been well described [4, 13–15, 19, 20]. Saile and colleagues suggest that IPV in conflict affected settings is perpetuated by a re-victimisation process among women and a victim-perpetrator transformation in men that leads to a normalisation and acceptance of violence [4]. Our findings encourage future studies that clarify the relationship between previous exposure to violence and high rates of IPV in conflict affected northern Uganda and other conflict affected settings.

Interestingly, when compared with the 2016 Uganda DHS [9] findings from the Acholi region of Uganda (where our study was conducted), the women in our survey reported markedly higher levels of IPV of all types. The DHS found a lifetime prevalence of IPV among women in the Acholi region of 59.9% compared with 78.5% of women in our survey. Sexual IPV rates were substantially lower in the DHS survey, which found a lifetime prevalence of 8.8% and 12-month prevalence of 6.3%, compared with 46.9 and 28.1% respectively among the women in Awach and Lalogi in our study. Rates of emotional abuse in our survey were nearly twice as high as those reported by the DHS [9]. There were also notable differences in education level between the Acholi women surveyed in the DHS and the women in our study: nearly three quarters of the women in our survey were unable to read, compared with 45% of women living in the Acholi region in the DHS survey. While nearly a third of Acholi-region women in the DHS had completed primary education or above [9], less than 15% of the women in our survey had obtained this level of education. The women in our survey were from areas heavily affected by internal conflict and violence over 20 years, and consequently schooling had been disrupted for many. However, while other studies from Uganda and SSA [7, 24, 25] have reported higher rates of IPV among women with lower levels of education, we did not find a significant association between education level and IPV in the logistic regression, suggesting that other factors contributed to the higher rates of IPV among the women in our study.

The strongest predictor of IPV in our logistic regression model was problematic alcohol use by the partner. Alcohol abuse by men has been found in numerous studies to be a significant determinant of IPV against women [4, 7, 16, 25, 27]. One third of the women in our study reported that their husbands were drunk every day or almost every day. These women were twice as likely to experience IPV compared with women whose husbands were not drunk almost every day. A similar proportion of daily drinkers was self-reported by men living in conflict affected Lira in northern Uganda [28], and these men were twice as likely to be heavy drinkers compared with men living in other, more peaceful regions of Uganda.

Alcohol use in Uganda has economic and cultural underpinnings. It is socially accepted in many settings and its consumption is unregulated [28]. There is a high level of unrecorded alcohol production and consumption, and many households are involved in brewing and distillation of alcohol. As such, alcohol production is the livelihood of many poor families [29]. Up to 10% of the population is dependent on alcohol, and research has indicated that political instability, poverty, and low levels of employment contribute to high levels of alcohol consumption [28]. Other studies from northern Uganda have described an association between high levels of alcohol use in men and past experiences of traumatic events [22, 30]. It is most likely that previous exposure to trauma contributed to the scale of the problem in the communities we surveyed, possibly through demoralisation and feeling helpless about the future. However, as exposure to conflict was not measured in the present study, we are unable to draw conclusions regarding an association between exposure to conflict and the development of substance use disorders. Our findings substantiate those from other studies that have reported an association between alcohol abuse and IPV, but further research into levels of alcohol consumption and the factors related to problem drinking in conflict affected northern Uganda is needed.

Controlling behaviours exhibited by partners are an early warning sign of heightened risk of IPV [9, 28]. Such behaviours include insisting on knowing their whereabouts, and controlling their access to friends and family. In our study, women who reported that their partners exercised controlling behaviours had an OR of 1.2 for IPV compared with women who did not report these behaviours, and this was significant when potential confounding factors, such as alcohol, were controlled for. The most frequently reported controlling behaviour exhibited by partners in our study was getting angry if the woman spoke to another man, reported by 67.4% of women. Nearly two thirds of women in our survey (64.2%) reported that their partner insisted on knowing where they were at all times, and 54.9% of women told us that their partner expected them to ask his permission before seeking healthcare for themselves. The risk of IPV has been shown to increase with the number of controlling behaviours exhibited [9, 33], and in our study the odds of experiencing IPV increased by 0.94 for each additional controlling behaviour. Nearly two-thirds of women surveyed (63.8%) reported experiencing at least 3 different controlling behaviour types by their partners, which was markedly higher than the findings from the 2016 Uganda DHS, in which 37% of women experienced 3 or more controlling behaviours [9].

In our study, younger women were significantly more likely to have experienced IPV within the past 12 months, although the effect was weak, with an adjusted OR of 0.95. An increased risk for IPV among younger women has been widely reported in SSA and globally [7, 16]. In a study on forced sexual debut among young women in low- and middle-income countries, East Africa had the highest reported prevalence of IPV among adolescents [26]. Many of the young women in Awach and Lalogi may have been exposed to additional recognised risk factors for IPV, such as childhood exposure to violence [4, 16], and a childhood spent in IDPs. Girls living in IDPs in Northern Uganda were at increased vulnerability for underage sex and coercion, with some engaging in ‘survival sex’ whereby sex was exchanged as a commodity for food or clothing [23]. In the conflict affected setting, an erosion of Acholi processes and belief systems that previously provided young girls with high levels of personal and sexual security has been described [20, 23].

In contrast to previous findings [7, 31], we found no association between IPV and women’s justification for IPV, although many women in our survey had attitudes that supported IPV in certain situations. Justification of IPV is common in Uganda [32]. In a community-based survey of more than 5000 women in the Rakai district of Central Uganda, 70% of men and 90% of women reported IPV to be acceptable in some situations such as if the woman refused to have sex with her partner or sought contraception without his permission [16]. Similar supportive attitudes are observed across SSA [33]. Whilst our findings indicate that among the women in our survey, justification of IPV did not confer an increased risk of IPV, the high level of acceptance reported by women is concerning and invites further research.

Under reporting of IPV is a global phenomenon, and a recognised feature of IPV in Uganda [19]. Durick describes a phenomenon whereby Ugandan women ‘suffer in silence’, without reporting IPV to family, friends, or authorities [19]. In their qualitative study, Annan and Brier found that structural barriers, including police corruption, sustain IPV in northern Uganda [22]. The Uganda DHS found that nearly a third of women suffering IPV in the Acholi region had never reported or told anybody about it [9]. The women in our study were socially isolated in their experiences: almost 85% of them reported that they did not think it was acceptable to discuss family matters with people outside the family, and 41.6% of women said that their partner tried to keep them from seeing their female friends. While not statistically significant, these findings are troubling and suggest that women suffering IPV in northern Uganda are unable to discuss their experiences with non-family members, perpetuating the culture of silence and making help-seeking less possible.

Our finding that male alcohol use is a key predictor of IPV in conflict affected northern Uganda suggests that interventions focused on reducing alcohol consumption and its related harms will be critical to reducing and preventing IPV in this region. There is strong evidence for screening and brief intervention for alcohol use disorder (AUD) in non-conflict settings, and guidelines on assessing AUD in conflict affected settings have been produced [34]. However, further evaluation of the feasibility and effectiveness of AUD interventions in conflict affected settings is urgently needed, together with greater support for the development and implementation of stronger alcohol regulation policies [35]. Addressing the factors behind the high rates of AUD among people affected by conflict (such as exposure to violence, dispossession, limited livelihoods, and a sense of despair) will also be critical to reducing the impact of AUD and preventing its emergence among younger generations. Interventions that recognise and treat mental health issues that underlie and are produced by AUD are also needed. Furthermore, opportunities and training to enable alternative livelihoods for those women involved in the production of illicit alcohol will be needed to facilitate a shift away from this economy and culture.

Another key implication of our findings is the seemingly entrenched attitudes that justify and sustain IPV in the communities we surveyed. Interventions to reduce and prevent IPV will need to support a change in attitudes and behaviours around gender based violence. Existing interventions for IPV in SSA have demonstrated that changes in attitudes and behaviours in IPV are possible through community-supported programs that use education and open discussion to break down taboos around IPV, HIV and gender [3]. Programs that have targeted boys and adolescents have been found to be more effective compared with those that target adult males only, and programs that have integrated education with HIV prevention services have led to reduced HIV exposure among women [3]. Given the high levels of engagement with health services among the women we surveyed, similar intersectoral approaches might be effective in conflict affected communities of northern Uganda. For example, an education-based IPV program that is integrated with health services could enable improved awareness of IPV while also offering screening and intervention for HIV and AUD, together with provision of condoms and other harm reduction measures. Further research into the acceptability and effectiveness of this type of approach in conflict affected northern Uganda is needed.

Our findings add to the limited body of research on factors associated with IPV in conflict affected northern Uganda. While the Uganda DHSs of 2011 and 2016 also examined the prevalence of IPV in the Acholi region of northern Uganda, they did not use logistic regression or other statistical analyses to elicit independently associated factors. Furthermore, the Uganda DHSs use a sampling frame to ensure proportional representation of each district in Uganda. This approach produces findings that are broadly representative from each region but may not allow for detection of findings unique to particular regions. For example, the 2016 Uganda DHS eliminated a particular cluster of the Acholi subregion in its sample selection due to land disputes. [9] This may have led to under-reporting of IPV for the Acholi district; the instability within communities that land disputes cause is a recognised risk factor for IPV. It is possible that the higher rates of IPV found in our survey reflect the impact of conflict on our population.

An important limitation of this study was that our study population comprised mainly of women living close to health centres. The women in our study were relatively well serviced by health facilities as reflected by higher rates of HIV testing and contraception use compared with women in the DHS survey [9]. Our findings may therefore not reflect the experiences of women living in more remote parts of northern Uganda. In addition, as with other studies and surveys on IPV, it is possible that there was under-reporting of experiences by our participants. We had conceived that the majority of women would be able to answer the survey on the tablets, hence improving privacy, but given the high level of illiteracy all surveys were administered by an interviewer. Whilst efforts were made to ensure that interviews were private, it is possible that women felt unable to fully disclose the nature of their experiences. Finally, while our research was conducted with women living in regions that were severely affected by conflict, we did not undertake an assessment of exposure to conflict, which prevents us from drawing conclusions regarding associations between this and the risk for IPV.

## Conclusions

This study has shown that IPV rates among women in rural, conflict affected regions of northern Uganda are extremely high, and has added to the small body of research on this topic. IPV in this region is underpinned by attitudes that normalise and justify it, and a culture of silence that dissuades women from seeking help. Younger women are at higher risk for IPV despite having improved educational and economic opportunities compared with older generations. They are likely to be affected by risk factors unique to conflict affected settings, such as childhood exposure to violence and displacement. As in other studies, alcohol abuse and controlling behaviours among male partners were shown to be highly prevalent, and strong predictors of IPV towards women. Changing the paradigm of high IPV rates in conflict affected northern Uganda will require an approach that recognises the influence of post-traumatic psychological issues, substance abuse, and gender power imbalances in sustaining IPV. The results of our study suggest that interventions to reduce alcohol consumption among men are likely to have important benefits in reducing levels of IPV, and invite further research into substance use in northern Uganda. Future studies are also needed to better understand the structural factors that sustain and normalise IPV within the conflict affected setting, so that appropriate preventive and responsive approaches can be developed.

## Data Availability

The datasets used and/or analysed during the current study are available from the corresponding author on reasonable request.

## Abbreviations

DHS: Demographic and Health Survey
HIV: Human immunodeficiency virus
IDPs: Internally displaced persons camps
IPV: Intimate partner violence
LRA: Lord’s Resistance Army
SSA: Sub-Saharan Africa
UNSW: University of New South Wales
WHO: World Health Organization

## References

[1] National Institute of Justice (NIJ). [Internet]. WA: NIJ; 2017. Intimate partner violence. Mar 30 2017 [cited Feb 3 2018]. Available from: https://www.nij.gov/topics/crime/intimate-partner-violence/pages/welcome.aspx

[2] Kitara D, Odongkara B, Anywar D, Atim P, Amone C, Komakech D (2012). Domestic violence in Gulu, northern Uganda. East Cent Afr J Surg.

[3] McCloskey Laura Ann, Boonzaier Floretta, Steinbrenner Sheila Young, Hunter Theresa (2016). Determinants of Intimate Partner Violence in Sub-Saharan Africa: A Review of Prevention and Intervention Programs. Partner Abuse.

[4] Saile R, Neuner F, Ertl V, Catani C (2013). Prevalence and predictors of partner violence against women in the aftermath of war: a survey among couples in northern Uganda. Soc Sci Med.

[5] United nations. Report of the fourth world conference on women. New York: United Nations; 1995 (A/ CONF.177/20/Rev.1) p 223.

[6] World Health Organization (WHO) (2015). Global and regional estimates of violence against women: prevalence and health effects of intimate partner violence and non-partner sexual violence.

[7] Abramsky T, Watts C, Garcia-Moreno C, Devries K, Kiss L, Ellsberg M (2011). What factors are associated with recent intimate partner violence? Findings from the WHO multi-country study on women’s health and domestic violence. BMC Public Health.

[8] Durevall D, Lindskog A (2015). Intimate partner violence and HIV in ten sub-Saharan African countries: what do the demographic and health surveys tell us?. Lancet Glob Health.

[9] Uganda Bureau of Statistics (UBOS) and ICF (2018). Uganda demographic and health survey 2016: key indicators report.

[10] Uganda Bureau of Statistcs (UBOS) and ICF International Inc (2012). Uganda Demographic and Health Survey 2011.

[11] Kenya National Bureau of Statistics, Ministry of Health/Kenya, National AIDS Control Council/Kenya, Kenya Medical Research Institute, National Council for Population and Development/Kenya, and ICF International. Kenya Demographic and Health Survey 2014. Rockville, MD, USA: Kenya National Bureau of statistics, Ministry of Health/Kenya, national AIDS control council/Kenya, Kenya Medical Research Institute, National Council for Population and Development/Kenya, and ICF International. 2015.

[12] Garcia-Moreno C, Jansen HA, Ellsberg M, Heise L, Watts CH (2006). Prevalence of IPV: findings from the WHO multi-country study on women’s health and domestic violence. Lancet..

[13] Dolan C (2009). Social torture: the case of northern Uganda, 1986–2006.

[14] Reliefweb [Internet]. Kampala: Advisory Consortium on Conflict Sensitivity (ACCS); 2013. Northern Uganda conflict analysis; September 2013 [cited 30 Jan 2018]. Available from: https://reliefweb.int/sites/reliefweb.int/files/resources/ACCS_Northern_Uganda_Conflict_Analysis_Report.pdf

[15] United Nations Development Programme (UNDP). Uganda human development report 2015. Kampala, Uganda: UNDP. p. 2015.

[16] Koenig MA, Lutalo T, Zhao F, Nalugoda F, Wabwire-Mangen F, Kiwanuka N (2003). Domestic violence in rural Uganda: evidence from a community-based study. Bull World Health Organ.

[17] Stark L, Roberts L, Wheaton W, Acham A, Boothby N, Ager A (2010). Measuring violence against women amidst war and displacement in northern Uganda using the “neighbourhood method”. J Epidem and Comm Health.

[18] Kouyoumdjian F, Calzavara L, Bondy S, O’Campo P, Serwadda D, Nalugoda F (2013). Risk factors for intimate partner violence in women in the Rakai community cohort study, Uganda, from 2000 to 2009. BMC Public Health.

[19] Durick H. Conflict affected development in northern Uganda: the importance of holistically addressing sexual and gender-based violence: University of Tennessee Honors Thesis Projects; 2013. Retrieved from http://trace.tennessee.edu/utk_chanhonoproj/1603

[20] Okello MC, Hovil L (2007). Confronting the reality of gender based violence in northern Uganda. Int J of Transitional Justice.

[21] Kwagala B, Wandera S, Ndugga P, Kabagenyi A. Empowerment, partner’s behaviours and intimate partner physical violence among married women in Uganda. BMC Public Health. 2013;13(1112). 10.1186/1471-2458-13-1112. 10.1186/1471-2458-13-1112 PMC4219526 24289495

[22] Annan J, Brier N (2010). The risk of return: intimate partner violence in northern Uganda’s armed conflict. Soc Sci Med.

[23] Patel S, Muyinda H, Sewankambo N, Oyat G, Atim S, Spittal P (2012). In the face of war: examining sexual vulnerabilities of Acholi adolescent girls living in displacement camps in conflict-affected northern Uganda. BMC Int Health and Human Rights.

[24] Jewkes R (2002). Intimate partner violence: causes and prevention. Lancet..

[25] Tumwesigye N, Kyomuhendo G, Greenfield T, Wanyenze R (2012). Problem drinking and physical intimate partner violence against women: evidence from a national survey in Uganda. BMC Public Health.

[26] Decker M, Latimore A, Yasutake S, Haviland M, Ahmed S, Blum R (2015). Gender-based violence against adolescent and young adult women in low- and middle-income countries. J Adolesc Health.

[27] Ebenezer S, Adjah O, Agbemafle I (2016). Determinants of domestic violence against women in Ghana. BMC Public Health.

[28] Tumwesigye N, Kasirye R, Obot I, Room R (2005). Gender and the major consequences of alcohol consumption in Uganda. Alcohol, gender and drinking problems: perspectives from low and middle income countries.

[29] Adelekan, M. Noncommercial Alcohol in Sub-Saharan Africa. WA: International Center for Alcohol Policies (ICAP); 2008. p. 3–16. ICAP Review 3. Retrieved from https://ec.europa.eu/health/archive/ph_determinants/life_style/alcohol/forum/docs/alcohol_lib15_en.pdf

[30] Roberts B, Ocaka K, Browne J, Oyok T, Sondorp E (2011). Alcohol disorder amongst forcibly displaced persons in northern Uganda. Addictive Behaviours.

[31] Ogland E, Xu X, Bartkowski J, Ogland C (2014). Intimate partner violence against married women in Uganda. J Fam Violence.

[32] Speizer I (2010). Intimate partner violence attitudes and experience among women and men in Uganda. J Interpersonal Violence.

[33] Uthman O, Lawoko S, Moradi T (2009). Factors associated with attitudes towards intimate partner violence against women: a comparative analysis of 17 sub-Saharan countries. BMC Int Health Hum Rights.

[34] UNHCR, & WHO (2008). Rapid assessment of alcohol and other substance use in conflict affected and displaced populations.

[35] Roberts B, Ezard N (2011). Why are we not doing more for alcohol use disorder among conflict-affected populations?. Addiction..

